# Early Acquisition of Neural Crest Competence During hESCs Neuralization

**DOI:** 10.1371/journal.pone.0013890

**Published:** 2010-11-09

**Authors:** Carol Lynn Curchoe, Jochen Maurer, Sonja J. McKeown, Giulio Cattarossi, Flavio Cimadamore, Mats Nilbratt, Evan Y. Snyder, Marianne Bronner-Fraser, Alexey V. Terskikh

**Affiliations:** 1 Sanford-Burnham Institute for Medical Research, La Jolla, California, United States of America; 2 California Institute of Technology, Pasadena, California, United States of America; City of Hope National Medical Center, United States of America

## Abstract

**Background:**

Neural crest stem cells (NCSCs) are a transient multipotent embryonic cell population that represents a defining characteristic of vertebrates. The neural crest (NC) gives rise to many derivatives including the neurons and glia of the sensory and autonomic ganglia of the peripheral nervous system, enteric neurons and glia, melanocytes, and the cartilaginous, bony and connective tissue of the craniofacial skeleton, cephalic neuroendocrine organs, and some heart vessels.

**Methodology/Principal Findings:**

We present evidence that neural crest (NC) competence can be acquired very early when human embryonic stem cells (hESCs) are selectively neuralized towards dorsal neuroepithelium in the absence of feeder cells in fully defined conditions. When hESC-derived neurospheres are plated on fibronectin, some cells emigrate onto the substrate. These early migratory Neural Crest Stem Cells (emNCSCs) uniformly upregulate Sox10 and vimentin, downregulate N-cadherin, and remodel F-actin, consistent with a transition from neuroepithelium to a mesenchymal NC cell. Over 13% of emNCSCs upregulate CD73, a marker of mesenchymal lineage characteristic of cephalic NC and connexin 43, found on early migratory NC cells. We demonstrated that emNCSCs give rise *in vitro* to all NC lineages, are multipotent on clonal level, and appropriately respond to developmental factors. We suggest that human emNCSC resemble cephalic NC described in model organisms. *Ex vivo* emNCSCs can differentiate into neurons in *Ret.k^-^* mouse embryonic gut tissue cultures and transplanted emNCSCs incorporate into NC-derived structures but not CNS tissues in chick embryos.

**Conclusions/Significance:**

These findings will provide a framework for further studying early human NC development including the epithelial to mesenchymal transition during NC delamination.

## Introduction

NCSCs have been well characterized in a number of model organisms [1], [2], [3], [4], including mouse, chicken, frog, and zebrafish [5], [6], [7], but little is known about the mechanisms of human NC specification, migration and differentiation.

In humans, the NC starts to migrate before neural tube closure, as early as embryonic stage 9, around the third to fourth week of pregnancy [8]. Multiple pathologies such as peripheral neuropathies, skeletal and nervous system disorders and pigment disorders stem from aberrant NC specification, migration or differentiation [9], [10], [11]. The derivation of human NCSCs from human embryonic stem cells (hESCs) will help to define the cellular and molecular mechanisms operating in human cells and facilitate the development of diagnostic and therapeutic strategies.

The manipulation of signaling molecules and pathways to direct ESC differentiation has been widely reported in the literature. Historically, the derivation of peripheral nervous system (PNS) cell types from mouse and primate ESCs has relied on co-culture with the mouse stromal line PA6 and late exposure to BMP4 [12] or, in the case of hESCs, co-culture with PA6 [13]. Similar to the work reported here, NC derivatives were obtained from bovine inner cell masses, after induction of differentiation by withdrawal of growth factors and supplementation with ascorbic acid [14]. NCSCs derived from hESCs after extensive passages and/or using a combination of stromal induction and the addition of BMP2 have been reported [15], [16]. More recently, the generation of a mixed population, depending on the cell densities, of central nervous system (CNS) neural progenitors and NC was also reported [17], [18]. The obligatory co-culture with PA6 or MS5 and the generation of only small numbers of some NC lineages (sometimes requiring prospective isolation of ∼0.1% of the cell population) are hurdles in all of the current differentiation protocols. Additionally, because NC competency is a transient phenomenon that appears to be established very early during gastrulation [19], the possibility exists that extensive *in vitro* amplification alters the properties of early NC cells.

We report that under conditions selectively promoting neuralization of hESCs towards dorsal neuroepithelial fate, NC competence is acquired much earlier than previously possible. Human ESC-derived NCSCs upregulate SoxE genes, Wnt and TGFβ signaling pathways associated with NC specification, maintenance, and migration [20], [21]. Using Sox10, a transcription factor playing a critical role in NC development [22], we identified a distinct population of Sox10-positive cells migrating from adherent hESC-derived neurospheres. These emNCSCs can differentiate into all NC lineages including PNS neurons and glia, smooth muscle myocytes, chondrocytes and melanocytes and respond to the well-known signals that block NC specification in other model systems [23]. Migratory NCSCs can colonize aganglionic embryonic gut cultures, where they differentiate into neurons. Finally, grafting into chick embryos demonstrates that hESC-derived emNCSCs contribute specifically to proper NC derivatives, differentiating into neurons and glia in the cranial ganglia, glia along nerves, mesenchyme and connective tissues in cranial regions.

## Methods

### Culture of human ES cells

An NIH-approved commercially available human ES cell line (H9, obtained from WiCell, Wisconsin) was used to generate neural precursors/putative NC cells *in vitro*. Undifferentiated hESCs were maintained on irradiated mouse feeder layers and Matrigel (final dilution1∶30; BD Biosciences, cat # 356231) in Knockout DMEM (GibcoBRL/Invitrogen, cat #10829-018) with 20% Knockout Serum Replacement (GibcoBRL/Invitrogen, cat #10828-028), non-essential amino acids (100 µM final; GibcoBRL/Invitrogen, cat #11140-050), L-glutamine (final 1 mM; GibcoBRL/Invitrogen, cat #25030-081), β mercaptoethanol (100 µM final; GibcoBRL/Invitrogen, cat #21985-023), and supplemented with bFGF (final 8 ng/ml; Sigma, cat# F -0291-25UG). hESCs were passaged every 7-10 days by separating cells manually or using collagenase IV (final 1 mg/ml; GibcoBRL/Invitrogen, cat #17104-019) diluted in KO DMEM. Medium was changed every day and differentiating colonies were manually removed.

### Generation of emNCSC from human ESCs

The differentiation protocol was previously described [24], except that day 5 spheres were plated on fibronectin. Briefly, collagenase IV passaged cells were resuspended in PBS and large clusters, not appropriate for neurosphere formation, were gravity pelleted and discarded. Clusters of hESCs that were left in suspension (about 50-100 cells/cluster) were transferred to a new tube, gravity pelleted and washed with PBS one additional time to remove all residual media and single cells. Cells were resuspended in neurosphere suspension (NS) medium: 1∶1 ratio of DMEM/F-12 Glutamax (GibcoBRL/Invitrogen, cat# 10829-018) and Neurobasal media (GibcoBRL/Invitrogen, cat# 11140-050), BIT 9500 (final 10%; StemCell Technologies Inc, cat# 09500), L-glutamine (final 1 mM; GibcoBRL/Invitrogen, cat# 25030-081),50 U/ml penicillin, 50 µg/ml streptomycin (Invitrogen, cat#15070-063), ) and B27 (final 5%; GibcoBRL/Invitrogen, cat# 17504-044) supplemented with insulin (final 5 ng/ml; Sigma, cat# I4011), nicotinamide (final 5 mM; Sigma, cat# N0636) supplemented with bFGF (final 20 ng/ml; Chemicon, cat# GF003) and EGF (final 20 ng/ml; Sigma, cat# E9644). Neurospheres were cultured for 5 days in non-adherent polypropylene dishes (Ted Pella, Redding, CA), changing the media every 2 days. Day 5 neurospheres were plated on fibronectin-coated (final 1 mg/ml) culture dishes in NS medium. EmNCSCs were passaged every 3–4 days with Accutase.

### Illumina Microarray and Q-PCR analysis

Total RNA was extracted using the RNeasy kit and 1 µg of total RNA was reverse transcribed using the Quantitect kit (Qiagen) according to the manufacturer's suggestions. About 500 ng total RNA per time point was used for cDNA synthesis followed by cRNA synthesis/amplification and labeling using the “Illumina RNA amplification” kit (Ambion). The labeled cRNA was hybridized to Human WG-6 sentrix BeadChip Arrays (Illumina) and scanned following the manufacturer's instructions. Analysis was performed using the algorithms included with the GeneSpring software. Gene expression changes greater than 2-fold; parametric test, variance equal, P value cut off  = 0.05; Volcano statistic analysis GeneSpring software were considered significant. For Q-PCR, GAPDH was used for normalization and the data were analyzed using the standard curve method. Microarray and Q-PCR were processed by the Burnham Institute's gene analysis core facility. Q-PCR was performed with SyberGreen master mix (Invitrogen) according to the manufacturer's recommendation. Primer sequences are provided in Table 1. Q-PCR was perfomed as follows: Initial denaturation: 10 min at 95°C; 40 cycles of denaturation: 30 sec at 95°C, annealing: 1 min at 56°C, extension: 30 sec at 72°C; and one final cycle of denaturation for 1 min at 95°C, annealing for 30 sec at 65°C and final denaturation for 30 sec at 95°C. Microarray hybridizations were performed on Illumina arrays and analyzed with Genespring software. All data are MIAME compliant and that the raw data has been deposited in a MIAME compliant database (GEO, accession number #*pending*).

**Table 1:** Primers used for Q-PCR of *PAX3.*

NCSC Q-PCR	
Pax3-F	GCATGTTCAGCTGGAAATC
Pax3-R	ATGCTGTGTTTGGCCTTCTT
GAFP-F	TCCTGGAACAGCAAAACAAGG
GFAP-R	GTAGTCGTTGGCTTCGTGCTT
GAPDH F	GAAGGTGAAGGTCGGAGTC
GAPDH R	ATGGGATTTCCATTGATGAC

### Immunohistochemistry

Cells were fixed with 4% paraformaldehyde in PBS for 15 minutes at room temperature and rinsed 3 times with PBS. Following fixation, cells were blocked in 3% BSA or 10% goat serum for 10 minutes, primary antibodies (Table 2) diluted in 3% BSA/PBS or 10% goat serum/PBS were then applied at the appropriate concentration and incubated either at room temperature for 1 hour or overnight at 4°C. Cells were then washed 3 times for 3 minutes, and incubated at room temperature with an appropriate fluorochrome-conjugated secondary antibody (1∶1000 diluted in 3% BSA/PBS). Cells were washed 3 times for 5 minutes and incubated for 10 minutes with Hoechst dye #33342 or Dapi to label cell nuclei.

**Table 2:** Antibody purchasing and dilution information.

p75	Alomone Labs	Rabbit	1∶200 IF/1∶300 facs
Sox10	M. Wegner	Mouse	Undiluted
HNK-1	M. Fukuda	Mouse	1∶30 IF
Map2	Sigma	Mouse	1∶500
GFAP	Chemicon	Rabbit	Pre-diluted
SMA	Sigma	Mouse	1∶500-1∶800
TH	Chemicon	Chicken	1∶100
Peripherin	Chemicon	Rabbit	1∶1000
BRN 3a	Chemicon	Rabbit	1∶100
Ki67	Novocastra	Rabbit	1∶500
CD73	BD	Mouse	1∶500
Nestin	Chemicon	Mouse	1∶500
HuNU AG	Chemicon	Mouse	1∶250
HuC/HuD	Invitrogen	Mouse	1∶250
HNK-1 (in ovo)	DSHB	Mouse	1∶100
Neurofilament	DSHB	Mouse	1∶50
PLP	AVES Labs	Chicken	1∶50
Pax6	Chemicon	Rabbit	1∶500
Pax3	Zymed	Rabbit	1∶200
Sox9	Chemicon	Rabbit	1∶200

### Clonal expansion of emNCSCs and lineage analysis

Clonal analysis was performed as described by the Studer group[15]. Briefly, cells were enzymatically dissociated after exposure to Accutase for 10 min. The cells were sedimented by centrifugationand the pellet was resuspended and filtered through 40 µm mesh. Filtered cells were counted and directly plated onto polyornithine (1.6 mg/ml ) + fibronectin (2 µg/ml) coated dishes at clonal densities (∼10 cells/cm^2^). Cells were grown in NS medium for 15 days followed by mitogen (bFGF and EGF) withdrawal and culture in NS medium supplemented with BDNF (10 ng/ml), NGF (10 ng/ml), GDNF (10 ng/ml), 1 mM dibutyryl cAMP, CNTF (10 ng/ml) and neuregulin (20 ng/ml) for 15 days. Cell were fixed in 4% paraformaldehyde for 10 minutes at room temperature and incubated with primary antibody overnight at 4°C, followed by 1 hour incubation with the secondary antibodies at room temperature.

### Addition of exogenous factors to manipulate NC specification or migration

BMPs, Wnts and retinoic acid have all been found to play roles in the specification and migration of NC in model organisms [20], [23], [25]. Neurospheres were cultured (suspended for 5 days with 3 days of adherent culture) in NS medium in the presence of 100 ng/ml Noggin (a BMP antagonist) or a conditioned medium from DKK1-producing 293T cells. Adherent cultures were then stained with the NC markers Sox10, p75 or HNK-1.

### *In vitro* derivation of NC-derived lineages and directed differentiations

For spontaneous differentiation, emNCSCs were maintained at confluency for up to 3 weeks in NS medium. Basic NS medium (NS medium without EGF, FGF, insulin or nicotinamide) was modified as previously described for directed differentiation [26], [27], [28], [29]; Briefly, for neuronal differentiation, 50 ng/ml BMP2 (R&D Biosystems), for neuroglial, 10% FCS/5 mM Forskolin (Sigma) and for smooth muscle myocytes 1 ng/ml TGFβ3 (R&D Biosystems) were supplemented to NS basic medium. To induce chondrogenic differentiation, 1×10^6^ emNCSCs were centrifuged at 800×g for 3 min. Pelleted cells were fed with chondrogenic medium (Lonza) supplemented with 10 ng/ml TGFβ3 (USBiological). Medium was changed every third day. Cultures were fixed after 4 weeks and chondrogenic differentiation was assessed by immunohistochemistry (Alcian blue).

### Prospective isolation by Fluorescence Activated Cell Sorting

At day 9 of emNCSC derivation the adherent neurospheres (composed of neuroepithelial rosettes) were mechanically removed and putative migrating emNCSCs were detached with Accutase and resuspended in 3% BSA/PBS for 20 minutes to block non-specific binding. Cells were then incubated with p75 (1∶300), HNK-1 (1∶300) or CD73 (1∶500) for 30 minutes. Cells were washed in 3 ml of PBS and resuspended in 3% BSA/PBS with appropriate secondary antibodies (1∶1000) for 30 minutes and washed with 3 mls of PBS before being resuspended in 1% BSA/PBS and incubated with PI (live/dead stain). Cells were sorted according to fluorescence (FACSVantageSE DiVa, BD Biosciences, San Jose) and data were analyzed with FlowJo (version 8) software.

### Injection of emNCSCs into organotypic fetal gut cultures

The intestine was isolated from day 10.5-11 *Ret.k^-^* embryos. Mice carrying the *Ret.k^-^* mutation were previously characterized and analysis of homozygous embryos has demonstrated that the aganglionic phenotype is due to an early defect of the enteric NC [30]. The method for organotypic embryonic gut cultures was previously described [31]. Briefly, E10.5- 11 *Ret.k^-^* mouse embryos were obtained from timed pregnant heterozygous matings. The developing gut was dissected and placed across a V-shaped notch cut into Millipore paper (0.45 µm, Black gridded type HAGB, Millipore, Bedford MA). Each gut segment suspended on filter paper was placed into a Tersasaki well (60-well plate, Disposable Products, Technology Park, SA, Australia) overfilled with media to cover the entire gut segment and cultured in 5% CO_2_ at 37° for 7 days. EmNCSCs were injected into the intestinal lumen and walls using a fire-polished glass needle connected to a mouth-injection tube. On average, 1×10^5^ cells were injected per gut piece. After 7 days, gut cultures were used for whole mount immunostaining and cryosectioning. Whole mount immunostaining for neurofilament was performed as previously described [32]. The *Ret.k* mice were housed in accordance with NIH guidelines and the Burnham Institute animal house rules.

#### *In ovo* transplantation

Fertile chicken eggs were incubated until somite stage 7 to 12. Eggs were windowed and embryos visualized using India Ink (1∶10). Pelleted emNCSCs (∼10,000 cells) were injected beside the cranial neural tube, in the pathway of migratory NC. Eggs were sealed and incubated for 1–5 days, at which time the embryos were fixed and cryosectioned [33]. Sections were incubated overnight with antibodies raised against: human nuclei (mouse IgG1, Chemicon MAB1281, 1∶250), early neuronal marker HuC/HuD (mouse IgG2b, Invitrogen A-21271, 1∶250 [34]) and HNK-1 (mouse IgM, DSHB, 1∶100) and smooth muscle actin (mouse IgG2a, Sigma A2547, 1∶800). Sections were then washed and incubated in conjugated secondary goat anti-mouse antibodies (all from Invitrogen): IgG1 Alexa 488, IgG2a Alexa 594 (both 1∶2000), IgG2b Alexa 633 and IgM Alexa 350 (both 1∶1000). Sections were imaged using Zeiss Axioskop plus microscope with AxioVision 4.6 software.

## Results

### Derivation of human emNCSC from hES cells

**Figure 1:**
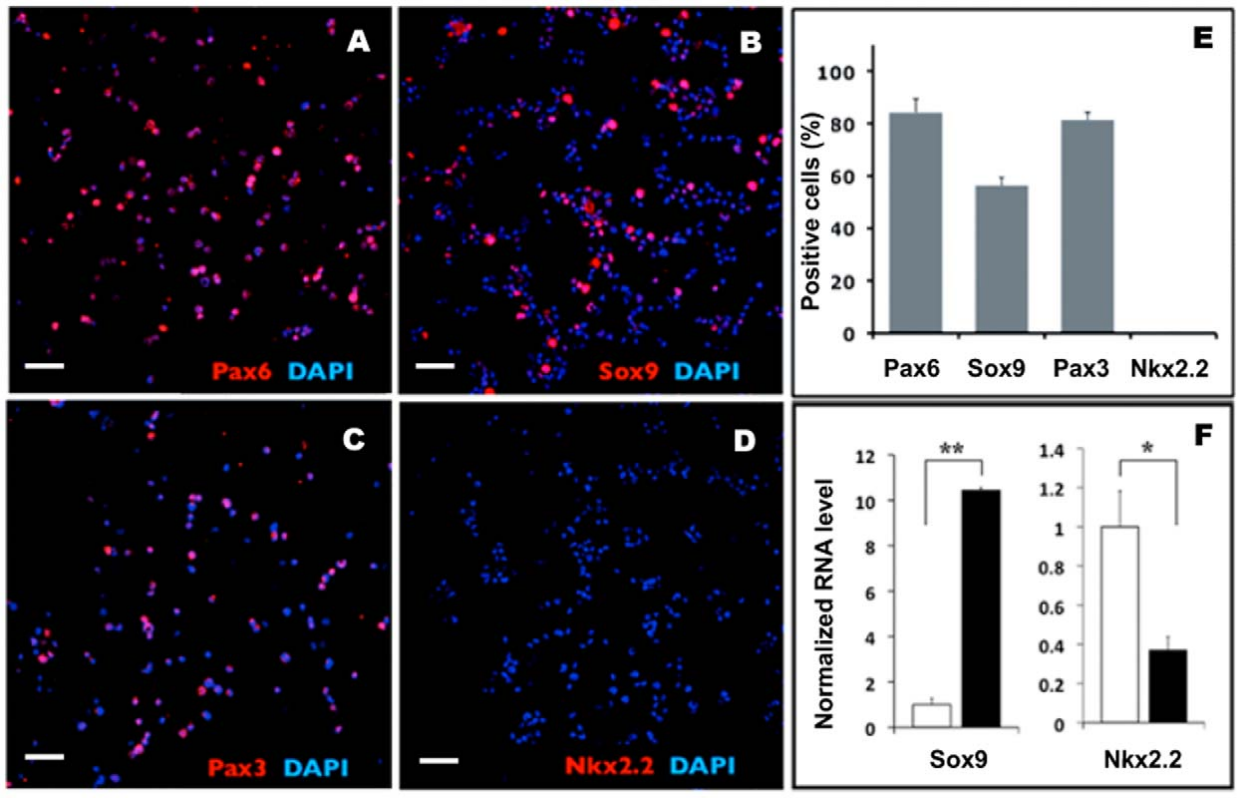
Neuralized human ES cell-derived express markers of dorsal neuroepithelium. hESCs were neuralized for 5 days in floating spheres, dissociated and cytospins were immunostained for Pax6 (**A**), Sox9 (**B**), Pax3 (**C**), and Nkx2.2 (**D**). (**E**) Quantification of the results in A–D. (F) Q-PCR analysis of Sox9 and NKX2.2 expression in day 5 spheres (black bars) *vs* hESC (white bars). All values normalized to 18S expression; * = p<0.05, ** = p<0.0001, one-tail unpaired t-test. Scale bars 50 µM.

hESCs can be efficiently differentiated into uniform neural progenitors, as previously shown [24]. Around day 5 of differentiation, rosette structures appeared within floating neurospheres that resemble primitive neuroepithelium and are reminiscent of neural tubes [14], [35]. The cells were collected and the dorsal – ventral identity of the cells was investigated (Figure 1). At this time of differentiation, most cells were positive for the pan-neuroepithelium marker Pax6 as well as dorsal neuroepithelial markers Pax3 and Sox9 but negative for the ventral neural tube marker Nkx2.2. The Q-PCR analysis was consistent with the immunostaining data. These results suggest that current neuralization conditions (see methods and expression analysis below) favor the derivation of dorsal neuroepithelium, which is the region of the neural tube from which NC arises *in vivo* [36], [37].

**Figure 2:**
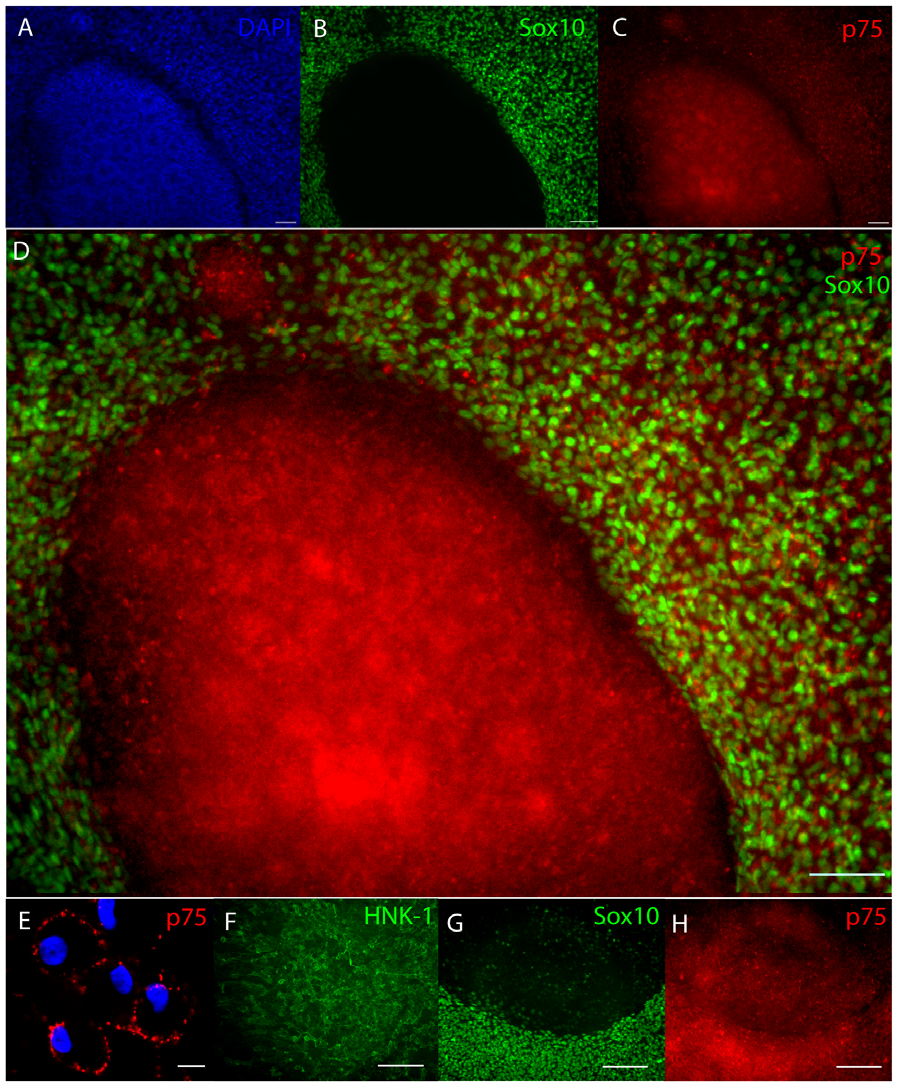
Early migratory NCSCs upregulate Sox10. Immunostaining of adherent neurospheres and emigrating NCSCs after 3 days on fibronectin-coated plates. (**A**) DAPI staining for nuclei. Note the rosette structures within the adherent neurospheres. (**B**) Sox10 staining is observed in emigrating cells, but not in the adherent neurospheres. (**C**) p75 staining is observed in adherent neurospheres and emigrating cells. (**D**) High magnification of the overlay in B–C. (**E**) Confocal image of emigrating NCSCs confirms the membrane localization of p75. (**F**) HNK-1 staining is observed in adherent neurospheres and emigrating cells. (**G, H**) Adherent neurospheres were manually removed and replated on fibronectin and the second wave of emigrating cells was observed. (**G**) Secondary emigrating cells acquire Sox10, while the majority of cells in the replated neurosphere are Sox10-negative. (**H**) The expression of in the second wave of emigrating cells. Scale bars in A, B, C and D represent 100 µm and 10 µm in E Bars in F,G and H depict 150 µm.

Primary murine NC cells are typically derived by plating neural tube explants from embryonic day 8.5 on fibronectin (a substrate permissive of NC migration *in vivo* [38]) and recovering the migrating cells [27]. Therefore, we plated day 5 neurospheres containing rosette structures on fibronectin and assayed NC marker expression after several days of migration. We found that 3 days of exposure to fibronectin resulted in a high number of migratory cells, which we called early migratory NCSCs. These cells express the NCSC markers Sox10, p75 and HNK-1 (Figure 2). The emNCSCs were uniformly positive for Sox10, while attached neurospheres composed of neuroepithelial rosettes (Figure 2D) were uniformly negative for Sox10 (Figure 2B, E). In contrast, both attached neurospheres and migrating cells were heterogeneously positive for the classical NC markers p75 and HNK-1 (Figure 2C, E, G). Because the adherent neurospheres resemble dorsal neuroepithelium, this observation appears similar to the *in vivo* situation. Indeed, in mouse embryos (E9.5-E11) at least some cells in the dorsal neuroepithelium (premigratory NC) are positive for the p75 marker [39] and human neural tube explants were reported to express p75 [40]. After an initial wave of NC emigration the adherent neurospheres (uniformly negative for Sox10) were scraped off the plate and replated. A second wave of emigrating cells was observed, which also acquired the Sox10 and p75 markers (Figure 2G–H). Most cells within the replated neurospheres remained Sox10-negative and the secondary wave of emigrating cells appeared to have higher levels of the p75 marker. These results suggest that many, if not all, cells within the adherent spheres are competent to upregulate Sox10 upon delaminating from neuroepithelial clusters and initiating migration on fibronectin. This behavior is reminiscent of an epithelial to mesenchymal transition (EMT), a characteristic feature of NC cells in model organisms [41].

### Emigration of emNCSCs from neuroepithelial clusters resembles EMT

**Figure 3:**
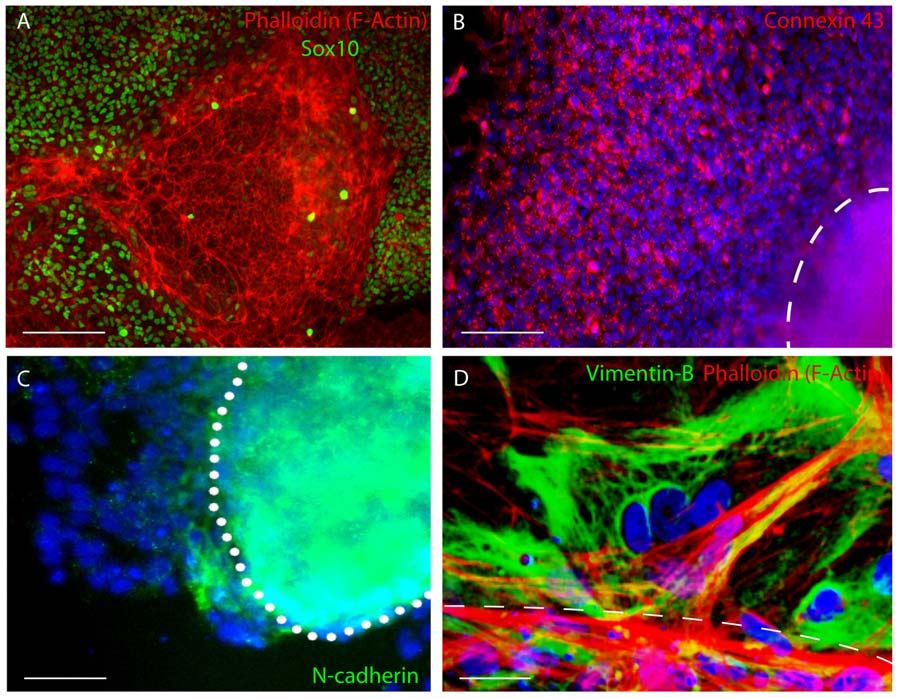
Epithelial to mesenchymal transition in migratory emNCSCs. (**A**) Phalloidin stains thick bands of f-actin within the adherent Sox10-negative neurosphere and thin cell border bands. Diffuse f-actin staining is visible in migratory Sox10-positive cells. (**B**) Connexin 43 (red) is acquired in migratory emNCSCs. Attached sphere is outlined. (**C**) N-Cadherin (green) is present in adherent neurospheres and gradually downregulated in migratory emNCSCs visualized by the nuclear DAPI IHC. (**D**) Confocal image of thick f-actin (red) bands at the border of an adherent neurosphere (outlined). Vimentin (green) is acquired in emNCSCs migrating away from the attached neurosphere. Scal bars in A and B represent 50 µm, in C 25 µm, and in D 10 µm. Cell nuclei were visualized by DAPI unless indicated differently.

Adherent neurospheres display thick bands of f-actin at the cell borders, characteristic of epithelial cells. Cells migrating from neurospheres acquire vimentin expression (Figure 3C–D) and have thin bands of f-actin at the cell borders and diffuse f-actin expression throughout the cell body (Figure 3A–B), consistent with observations in mesenchymal cells [42]. This alteration of the f-actin distribution between the rosette cells and the migrating cells is reminiscent of the EMT observed in NC cells delaminating from the neural tube *in vivo* [41]. Similarly, the adherent neurospheres are positive for N-cadherin and emNCSCs downregulate N-cadherin expression, in further support of an EMT [43]. In addition, emNCSCs upregulate the expression of gap junction protein connexin 43 (Cx43) at the onset of migration (Figure 3F). The expression of Cx43 was found to be critical for NC cell survival and characteristic of early migratory NC cells *in vivo* [44]. In summary, the transition from adherent neurospheres (further referred to as neuroepithelial clusters) to migratory Sox10-positive cells has many features of classical EMT observed during delamination of NC *in vivo* in model organisms.

### Microarray analysis of neuroepithelial clusters and migratory NCSC

**Figure 4:**
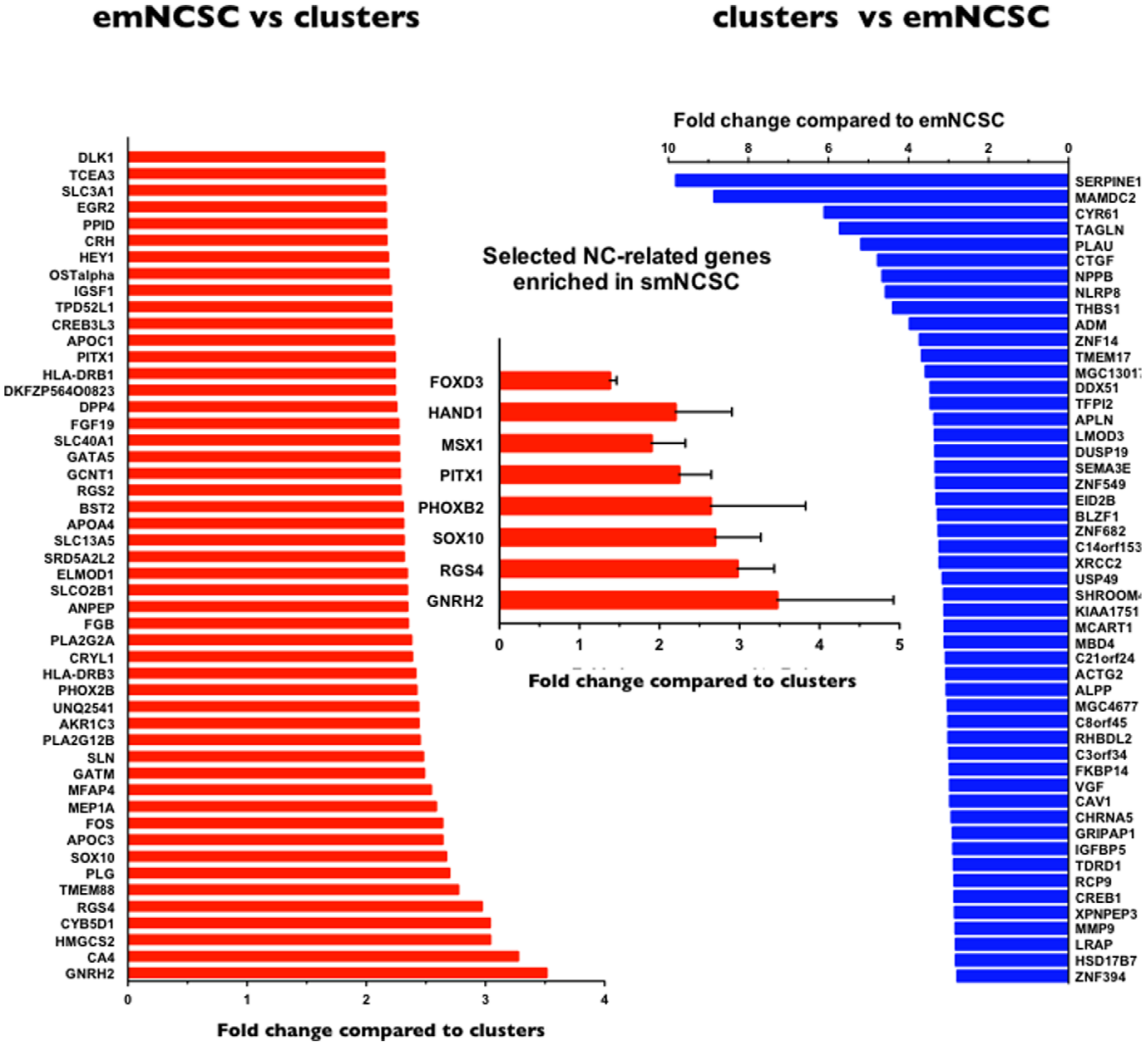
Microarray analysis of the adherent neurospheres and emNCSCs. The top most upregulated gene transcripts in emNCSCs (red) and neuroepithelial clusters (blue) are shown. The middle insert highlights the selected set of NC-related genes upregulated in emNCSCs.

The above results provide some distinctions between the Sox10-positive cells in neuroepithelial clusters and the Sox10-positive migratory cells. To further investigate the molecular difference between the two populations, we mechanically separated adherent neurospheres from migratory cells and performed microarray analysis of gene expression (Figure 4). We found that in migratory cells, 84 genes were significantly upregulated and 103 were downregulated compared to the neuroepithelial clusters. Confirming the immunostaining data, the *SOX10* transcript was upregulated in migratory cells. Several well-known regulators of the NC lineage were selectively upregulated in migratory cells (*e.g.*, *PhOX2b, RGS 4, HEY1, DLK1*, and *GnRH2*). Several other NC genes such as *FOXD3, MSX1, PITX1*, and *Hand1* were upregulated in migratory cells. Curiously, *IGFBP5*, a negative regulator of NC and a potential regulator of craniofacial skeletogenesis [45], was selectively downregulated in migratory cells, suggesting a potential interaction between the neuroepithelial clusters and emigrating cells.

In addition to the expression analysis described above, we determined the dynamic expression of key NC-related genes over 10 days of neuralization using the entire cultures (Figure S1). Among the upregulated transcripts were the growth factors *Wnt3a* (2-fold at 72 hours), *Wnt1* (2-fold at 96 hours) and *BMP4* (2-fold by day 3) as well as the downstream transcription factors *Sox9* (2-fold at day 3), *Sox10* (7-fold day 6), *ZIC1* (2-fold at day 3), *MSX1* (2-fold by day 2) and *SNAIL*, (2-fold by 60 hours). In addition, we used Q-PCR to examine the expression of *Pax3*, one of the earliest gene defining the NC field [46]. *PAX3* transcripts were found to be upregulated 2-fold at day 3 and 10-fold at day 5 of hESC neuralization.

We demonstrated a dynamic regulation of most NC-related genes during hESC differentiation towards emNCSC, including the upregulation of many NC-related genes in emNCSC when compared to the neuroepithelial clusters. These data contribute to our understanding of gene regulatory network orchestrating neural crest formation and provide support to our interpretation of the *in vitro* human model system - the cells within the clusters are similar to the dorsal neuroepithelium, while emigrating cells resemble NCSCs.

### p75 expression does not discriminate emNCSCs from neuroepithelial clusters

**Figure 5:**
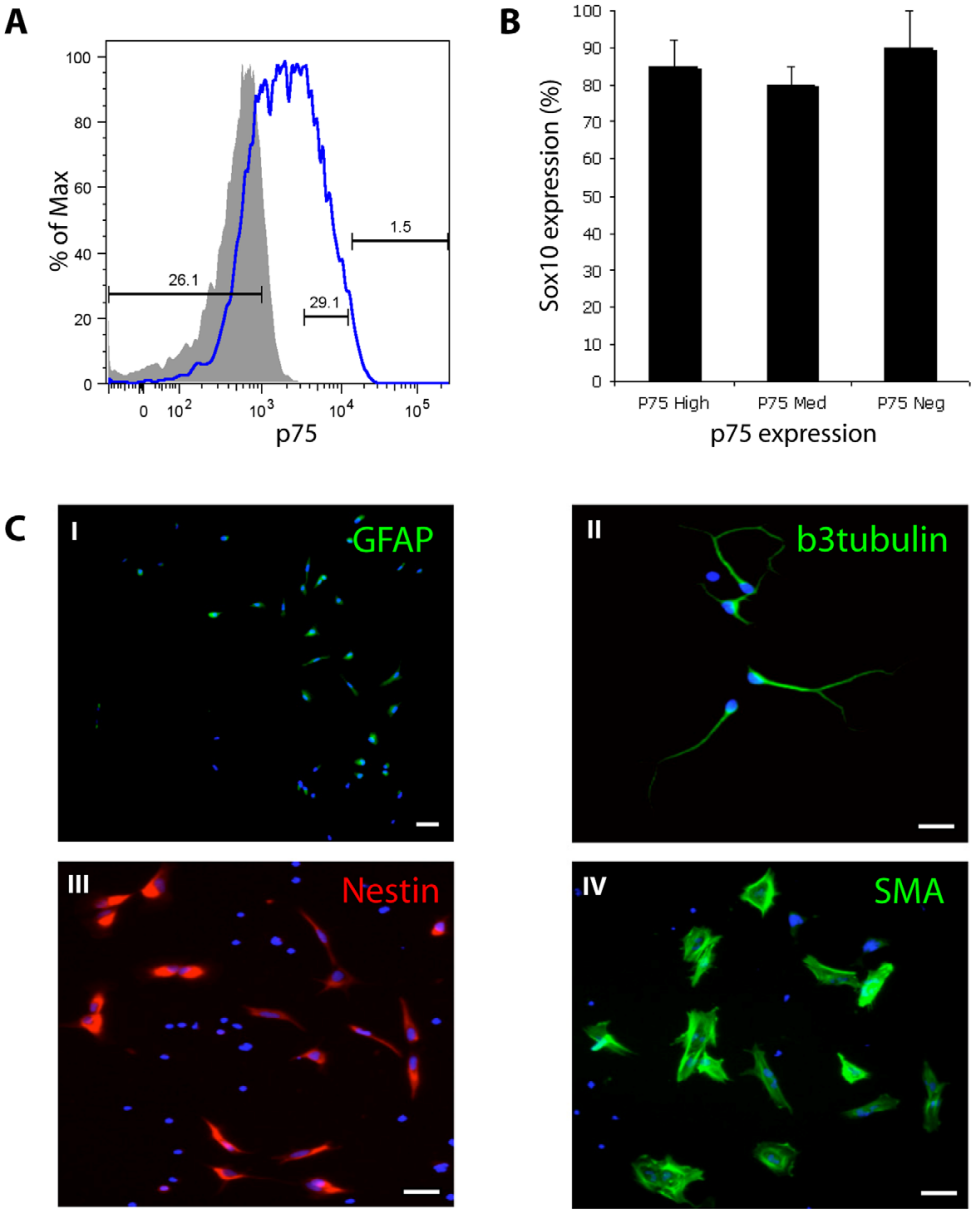
p75 expression doesn't discriminate emNCSCs from neuroepithelial clusters. (**A**) Day 8 cultures (containing emNCSC and neuroepithelial clusters) can be FACS-sorted based on p75 expression defining three populations: negative, medium and highly positive for p75. The grey profile corresponds to control situation with the secondary antibody only. (**B**) All fractions of cells were positive for Sox10 (85, 70 and 60% respectively for high, medium and negative). (**C**) All fractions can differentiate into neuronal, glial and smooth muscle lineages (p75-negative fraction is shown as an example). p75-negative sorted cells are reactive for; **I**. GFAP, **II**. β-3-tubulin, **III**. nestin, **IV**. smooth muscle actin. Cell nuclei are visualized by DAPI. Magnifications are 100x. Scale bars 30 µM.

Previously, the p75 marker was used for prospective isolation of NCSCs from rat embryo sciatic nerves [1] and when differentiated from hESCs after prolonged differentiation on stromal feeders [15]. We found that the membrane-specific immunostaining for p75 was present in both clusters and emigrating cells (Figure 2C–E). We employed flow cytometry to prospectively isolate cells from hESC neuralization cultures at day 8, based on p75 expression. Consistent with the immunostaining data, most cells were positive for p75 (Figure 5A). Cells expressing high, medium, and low levels of p75 were isolated by FACS and their Sox10 levels and differentiation potential were examined (Figure 5). Most cells, whether p75 high, medium, or low, expressed Sox10 (85%, 80%, and 90% respectively, Figure 5B). After one week of spontaneous differentiation on fibronectin young neurons (βIII-tubulin), glia (GFAP), smooth muscle cells (SMA), as well as undifferentiated cells (nestin) were found in all cultures (Figure 5C and data not shown). No selectivity for differentiation into any particular lineage could be detected in the three populations. Another marker, HNK-1, was previously proposed as a marker of NC in chick embryos [47] and was used in the hESC system [15]. Flow cytometry analysis of day 8 cultures revealed that over 90% of cells are positive for the HNK-1 marker (data not shown). These results suggest that the p75 and HNK-1 markers, previously used to prospectively isolate human NCSCs after prolonged differentiation on stromal feeders [15] are ineffective in discriminating migratory cells from the neuroepithelial clusters in our cultures. Because many (if not all) cells in the clusters can give rise to the migratory Sox10+ cells (e.g. when re-plated on fibronectin) it is possible that these pre-migratory NC cells already upregulated the p75 and HNK1 molecules.

### Differentiation of emNCSC into the NC lineages

**Figure 6:**
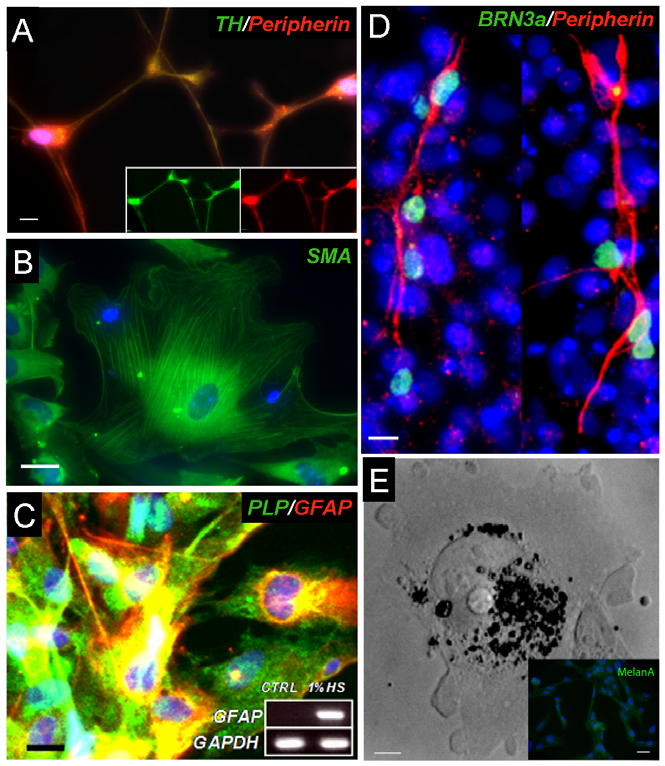
Spontaneous differentiation of emNCSCs. (**A**) Peripheral neuron-like cells are co-stained with TH (green) and Peripherin (red). Individual channels are shown in the inserts.(**B**) Smooth muscle cells reactive to smooth muscle actin. (**C**) The addition of 1% horse serum to the NS medium yielded GFAP-positive cells co-expressing p75NTR and proteolipid protein (PLP), consistent with Schwann cell identity. Inset, PCR detection of GFAP transcript. CTRL  =  NS medium. Scale bar  = 25 ìm. (**D**) Brn3a (red) and Peripherin (green) staining indicates differentiation into peripheral sensory-like neurons. (**E**) Bright field image of black inclusion bodies in a characteristic melanosome, inset box shows Melan A (green) reactivity. Nuclei were visualized by DAPI. Scale bars represent 40 µm in **A, C** and **D**, 60 µm in **D,** and 100 µm in **E** and its inset.

We determined whether emNCSCs as a population were able to give rise to a variety of NC lineages found *in vivo*, including neurons, glial or mesenchymal lineages, melanocytes, and adipocites. EmNCSCs were allowed to spontaneously differentiate in dense cultures through contact inhibition and were assayed for NC derivatives after 2–3 weeks (Figure 6 and Figure S2). After ∼2 weeks in culture, peripheral autonomic lineages were identified by co-staining of TH and peripherin [48] (Figure 6A) and sensory peripheral lineages were detected by BRN3a (premigratory crest and neurons of the DRG) reactivity (Figure 6B). Neuronal derivatives were found to comprise 30% ±10% of derivatives. Myocytes were identified by smooth muscle actin staining after one week in culture (Figure 6C), and were also observed to comprise approximately 30% ±10% of derivatives. Consistent with events during normal developmental and in agreement with the previous observations [15], neurogenesis preceded gliogenesis. Characteristic markers of Schwann cells could be detected in cultures after emNCSCs were passaged for 3–4 weeks and differentiated for additional two weeks (Figure 6D). Glial derivatives comprised 20% ±10% of total derivatives. Melanocytes (comprising 5% ±3% of derivatives) were observed after 3-4 weeks in culture. Melanocytes were identified by their characteristic black melanosome inclusions and Melan A reactivity (Figure 6E).

**Figure 7:**
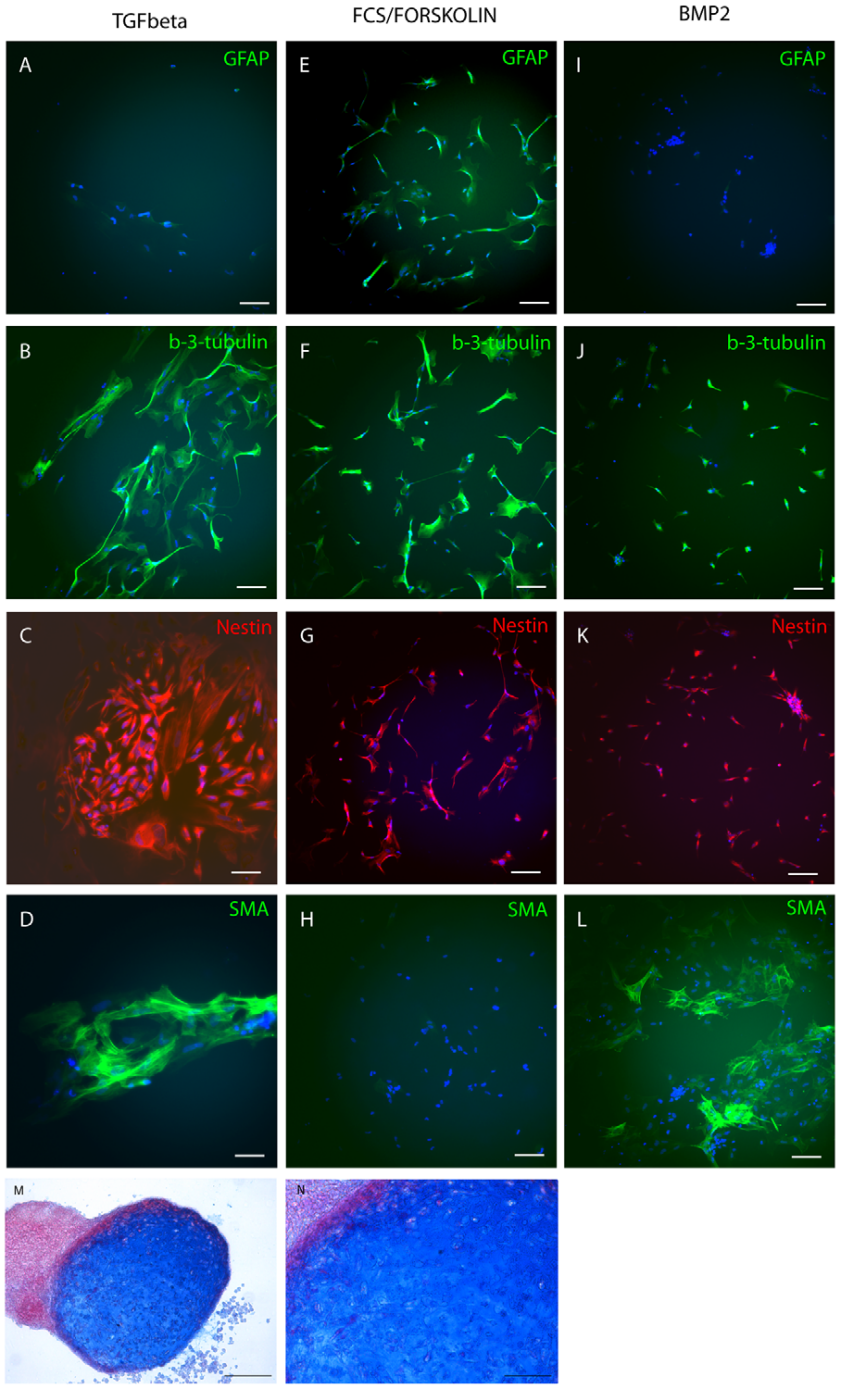
Directed differentiation of emNCSCs. The growth factors FGF, EGF, insulin, and nicotinamide were withdrawn from NS medium and the base medium is supplemented with TGFβ (**A-D**) to enrich differentiating cells for neuronal fates, FCS/Forskolin to enrich for neuro-glial fates (**E–H**) and BMP2 to enrich for smooth muscle (**I–L**). A, E and I show GFAP immunohistochemistry (green, astrocytes). B, F and J indicate bIII tubulin (green, immature neurons). C, G and K show nestin (red, neuroblasts) and D, H and L show SMA reactivity (green, smooth muscle actin). Cell nuclei were visualized by DAPI, bars in A–L represent 100 µm. (**M,N**) Chondrocytic differentiation can be directed in emNCSC cultures (see materials and methods for details) and cryosectioned pellets reveal collagen II by Alcian blue staining. Scale bars 100 µM in M and 25 µM in N.

Previously, NCSCs were observed to respond to various combinations of factors, selectively generating specific NC lineages [15], [26], [49]. The addition of single growth factors to NS basal medium (no growth factors) led to enrichment of distinct cell types in the cultures. Addition of TGFβ initiated mainly two cell types: neurons and myocytes at a ratio of 70% ±5%: 28% ±4% (Figure 7A–D, TGFβ). To enrich for neuro-glial precursors, 10% fetal calf serum and forskolin (5 µM) were added to NS base medium. Under these conditions, glia comprised 30% ±2% of the cells, neuronal markers were detected in 60% ±5% of the cultures and no SMA-positive cells were detected (Figure 7E–H, FCS/Forskolin). Culturing emNCSCs with BMP2 enriched for a neuronal phenotype (Figure 7I–L, BMP2). Fifty percent ±7% of cells were positive for β III tubulin. Five percent ±2% were positive for GFAP, indicating glial differentiation and another 40% ±3% stained positive for SMA, indicative of myocyte differentiation. For chondrocytic differentiation, emNCSCs were incubated in defined medium, in a high-density culture, under low-oxygen conditions for 4 weeks [27]. Comparable to data published earlier, this method resulted in cell aggregates that when sectioned, showed strong expression of collagen in the intercellular space as shown by Alcian blue staining (Figure 7M and N).

In summary, spontaneous and directed differentiation of human emNCSCs *in vitro* into multiple NC-specific lineages demonstrates their broad potential and ability to respond appropriately to developmental cues.

### Clonal expansion of emNCSCs and lineage analysis

**Figure 8:**
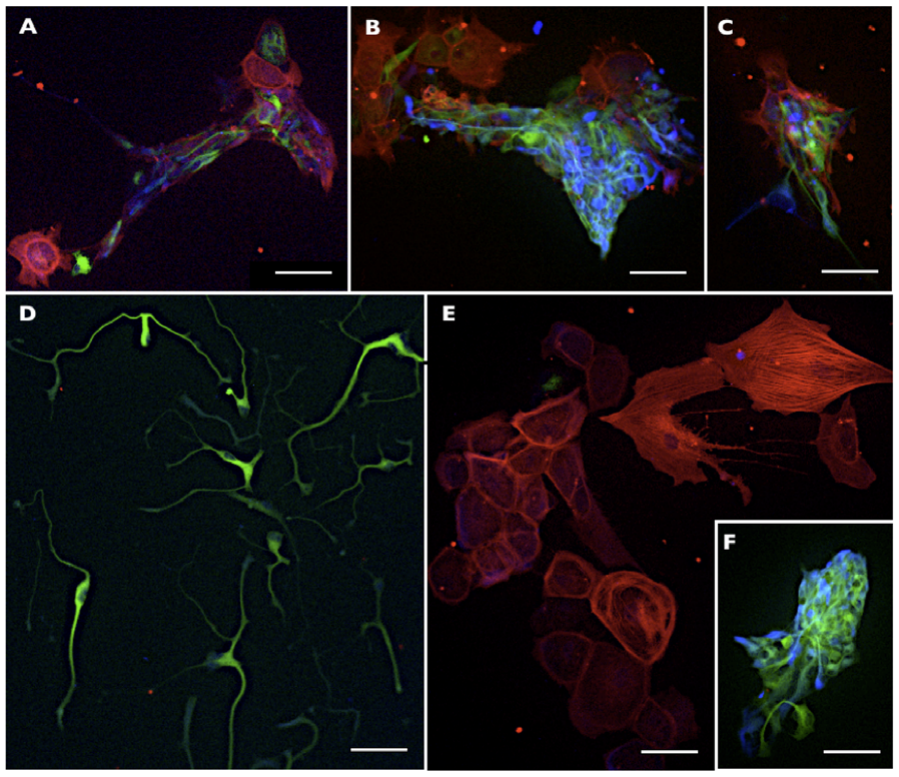
Clonal analysis of emNCSC. Representative examples of multipotent (**A–C**), bi-potent (**F**), and unipotent (**D, E**) clones of emNCSC. For all images: TuJ1 (green), SMA (red), GFAP (blue); Scale bars 50 µM.

Experiments in model organisms suggested that *in vivo*, NC cells include multipotent, oligopotent and unipotent clones [50], [51]. In order to assess the differentiation potential of emNCSCs at a single cell level, we performed clonal analysis experiments by plating single cell dissociated *emNCSCs* at clonal densities as previously described by the Studer group[15]. Staining of 20 clones for markers of neurons (TuJ1), glial cells (GFAP) and smooth muscle cells (SMA) revealed 12 clones (63%) positive for all three lineage markers, 3 clones (16%) positive for TuJ1 and GFAP, 3 clones (16%) positive for SMA only, and 1 clone (5%) positive for TuJ1 only (Figure 8). These results suggest that emNCSCs are multipotent on the clonal level.

### Inhibition of BMP and Wnt signaling re-specifies emNCSCs into CNS cells

**Figure 9:**
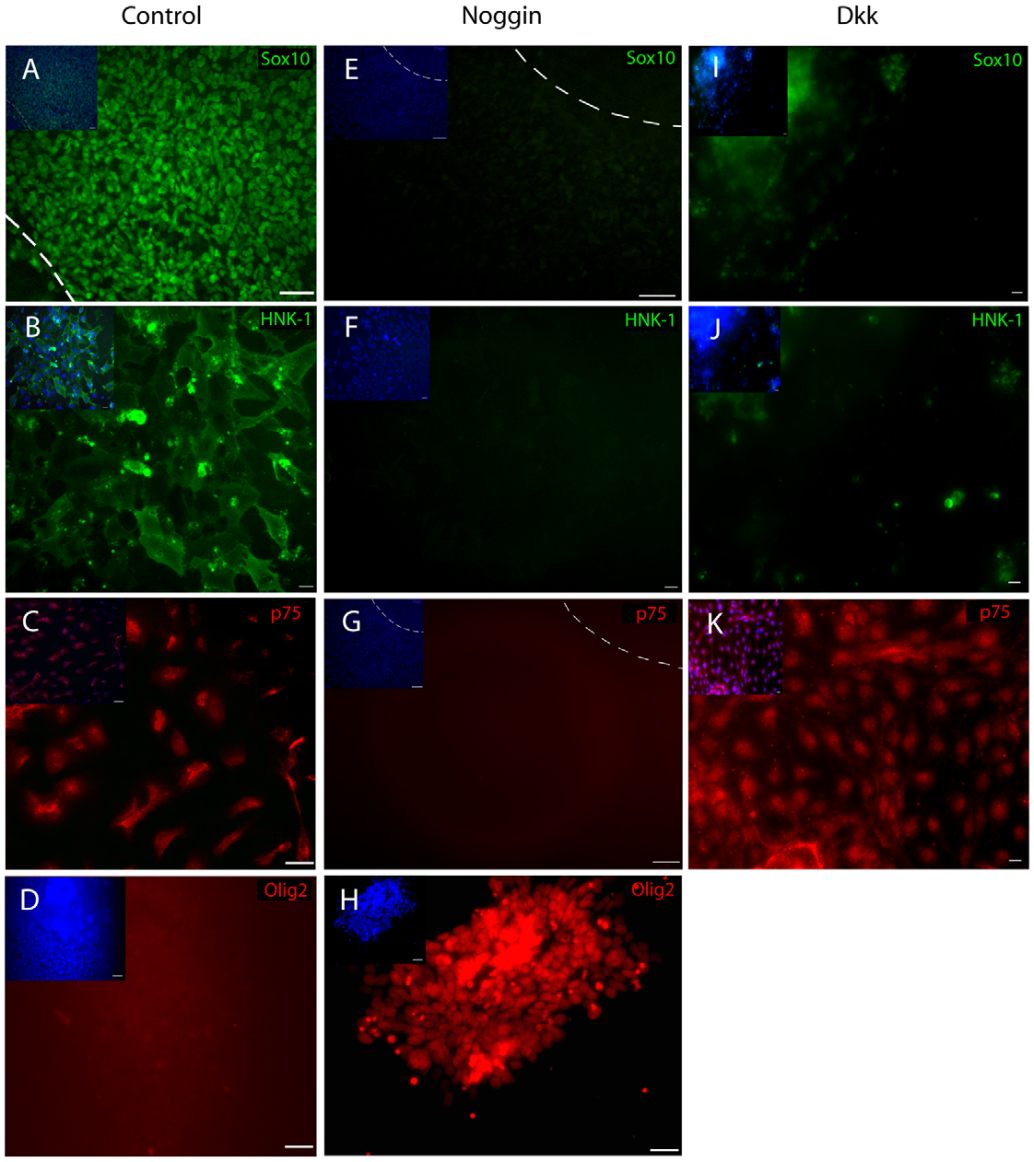
NC competency is ablated by treatment with Noggin or DKK. The effect of Noggin (BMP inhibitor) and DKK (Wnt inhibitor) on hESC-derived emNCSCs. Untreated (control) neurosphere cultures are strongly reactive for (**A**) SOX10 (green), (**B**) HNK-1 (green), and (**C**) p75 (red) and negative for the CNS marker (**D**) Olig2 (red). The addition of Noggin to NS medium ablates (**E**) SOX10 (green), (**F**) HNK-1 (green), and (**G**) p75 (red) and induces expression of the CNS marker (**H**) Olig2 (red). Culturing neurospheres from day 0 in media that was collected from 293T cells transfected with a DKK plasmid ablates expression of the NC markers (**I**) SOX10 and (**J**) HNK-1 while the low affinity neutrophin receptor (**K**) p75 was still expressed. The insets in upper left corners show the cell nuclei (DAPI). Scale bars represent 50 µm in A, and C–H, 10 µm in B, and 100 µm in I–K.

Exogenous factors, such as BMPs and Wnts are well-known modulators of NC specification and migration *in vivo* [20], [23], [52]. BMP4 is expressed in the avian dorsal neural tube at the time of neural tube closure when NC migration starts. Addition of Noggin at this stage, but not at later stages, inhibits NC formation and migration [23]. We tested the effect of Noggin on the derivation of emNCSCs from hESCs. Control cells show Sox10, HNK-1 and p75 reactivity (Figure 9A–C). The addition of Noggin (100 ng/ml, starting on day 0, medium was changed every other day) nearly ablated expression of the NC markers Sox10, p75 and HNK-1 (Figure 9D–F). At the same time, the migration of cells from each adherent neurosphere was less than in controls (70% of control, Figure 9J). Similarly, the addition of the Wnt/β-catenin antagonist Dkk1 ablated expression of the NC markers Sox10 and HNK-1, but not the low affinity neurotrophin receptor p75. The retention of p75 is consistent with the finding that Wnt signaling does not change the axial identity of cells in the developing neural tube, but rather re-specifies the cell fates at the early stages of NC development [53]. These results indicate that human emNCSCs appropriately respond to the stage-specific developmental signals, consistent with the early migratory nature of the emNCSCs identified in this study.

### Mesenchymal potential of emNCSCs

**Figure 10:**
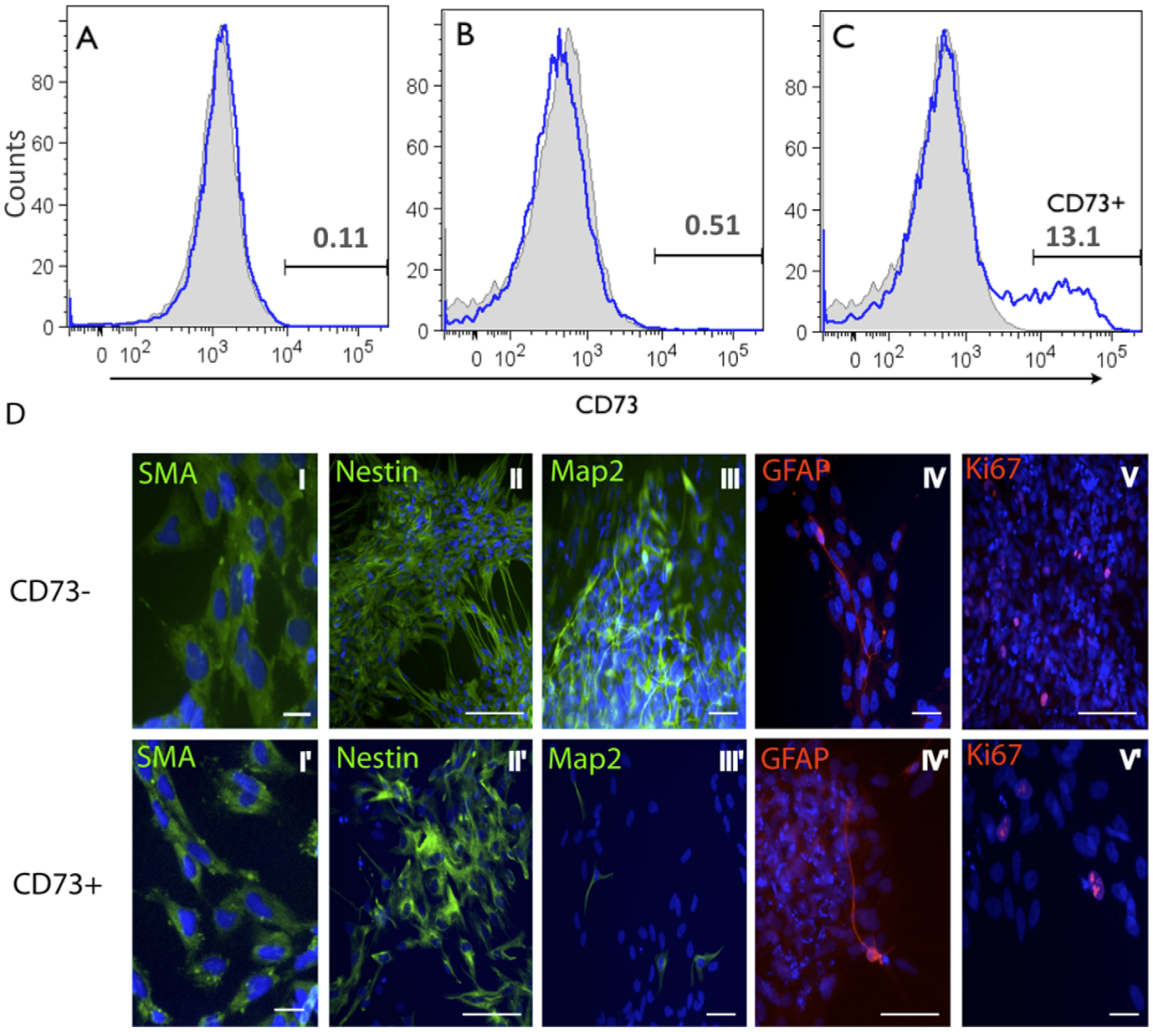
CD73 expression in emNCSCs. Early migratory NC cells are considered to be a uniform population of cells capable of differentiation into mesenchymal and PNS derivatives. To evaluate the potential of hESC-derived emNCSCs the mesenchymal marker CD73 was investigated at several time points, followed by prospective isolation and differentiation. (**A**) Day 5 neurospheres were tested for CD73 expression, approximately 0.11% of cells were positive. (**B**) Adherent neuroepithelial clusters were mechanically separated from migrating cells and tested for CD73 expression, approximately 0.5% of adherent neuroepithelial rosettes cells were positive. (**C**) 13% of cells migrating from neuroepithelial rosettes (SOX10-positive cells) are positive for CD73. (**D**) Migrating cells were sorted based on CD73 expression and cultured for 1 week. Both CD73-positive and -negative cells yielded nestin- and SMA-positive cells (areas with abundant staining are shown), however, the MAP2 reactive cells were enriched 10-fold in the CD73-negative fraction. Both populations had rare GFAP-positive cells and scattered KI67-positive cells. Scale bars represent 10 µm in I, I′ and V′, 50 µm in III, IV, III′ and IV′, and 100 µm in II, V and II′.

*In vivo*, early migratory NC cells (i.e. cephalic NC) are considered a uniform population of cells, capable of differentiation into mesenchymal lineage (*e.g.,* myocytes) and peripheral neurons and glia [51], [54]. In a previous study, human ESC-derived NCSCs were found to contain a small population of mesenchymal precursors, identified by the CD73 marker [15]. We examined CD73 expression in day 5 neurospheres prior to culturing on fibronectin, as well as in epithelial clusters and migratory cells 3 days post plating on fibronectin. The day 5 neurospheres displayed no CD73 as expected for undifferentiated cells of neural origin (Figure 10A). At day 8 post plating, neuroepithelial clusters were also negative for CD73, however, ∼13% of the migrating emNCSCs were positive for CD73 (Figure 10B–C). To investigate their differentiation potential, CD73-positive and -negative populations were isolated by FACS. Both populations were allowed to differentiate for one week after sorting and assayed for SMA, nestin, MAP2 and Ki67 markers. Both CD73-positive and -negative sorted cells gave rise to abundant SMA^+^ and nestin^+^ cells. GFAP^+^ cells were rare in both cases; however, CD73-negative cells produced approximately 10-fold more MAP2-positive cells than the CD73-positive fraction (Figure 10D). These data suggest that CD73-positive cells are much less efficient at differentiation into the neuronal lineage, as expected for the mesenchymal component of NC. Further experiments are needed, however, to demonstrate the selective advantage CD73-postive cells for mesenchymal differentiation.

### Injection of emNCSCs into organotypic embryonic gut cultures

**Figure 11:**
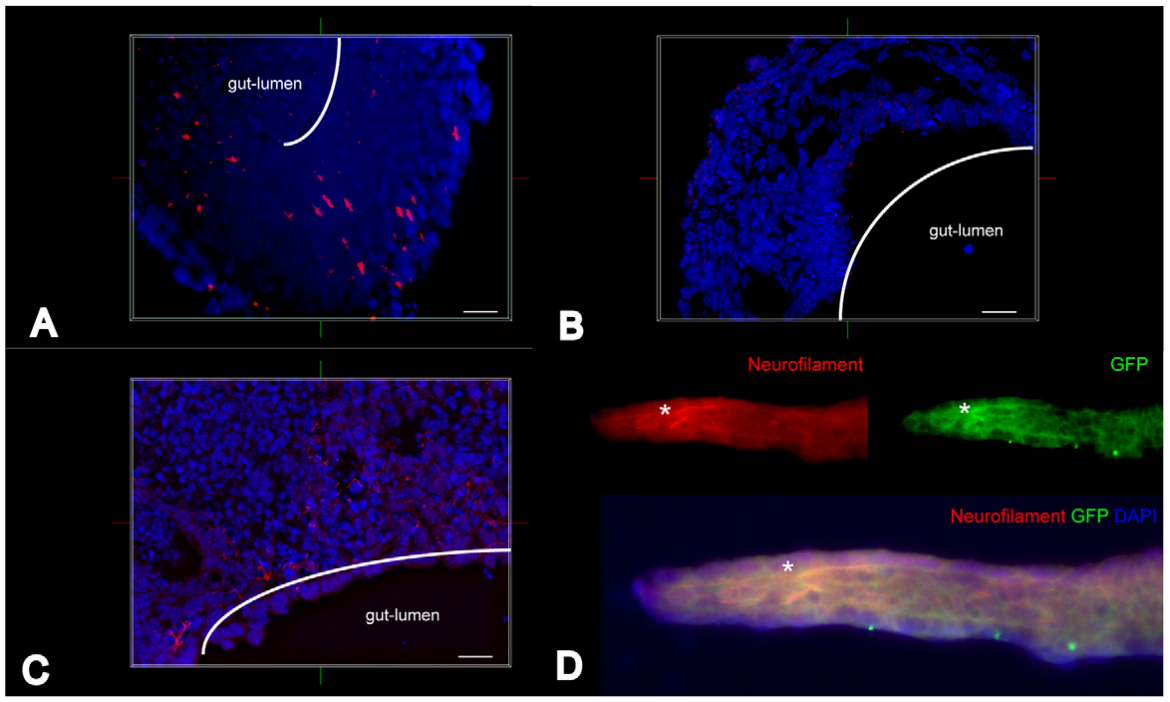
Injection of emNCSCs in aganglionic bowels in *ex vivo* organotypic cultures. EmNCSCs can colonize aganglionic guts in organotypic gut cultures and differentiate into neurons. (**A**) Confocal image of a cryosectioned *Ret* KO gut segment 7 days post injection labeled with fluorescent antibodies against neurofilament (red). (**B**) Non-injected *Ret* KO gut segment 7 days post injection showing no neurofilament (red) reactivity. (**C**) In heterozygous animals endogenous neurons are present in the non-injected gut cultures (neurofilament staining in red). Cell nuclei are visualized by DAPI. Scale bars in A-C,10 µm. (**D**) Whole mount of a *Ret* KO gut segment stained with a neurofilament marker (red) and anti-GFP expressed by emNCSC (green). The GFP staining overlaps with the neurofilament staining in several places showing emNCSC-derived neurons; one example is marked by asterisk. Single channels (red, neurofilament and green, GFP) are depicted on top of the merged photograph for better identification (Magnification 20x).

The ability of emNCSCs to colonize and differentiate into appropriate NC derivatives in a disease model was tested by injection of emNCSCs into aganglionic embryonic gut cultures derived from mice carrying *Ret.k^-^* mutation. As previously described, only NC-derived enteric neurons can repopulate the *Ret.k^-^* intestine [31]. Undifferentiated emNCSCs derived from hESC engineered to express GFP were injected into the knockout gut segments. After 7 days of culture, the tissue was cryopreserved, sectioned and immunostained for GFP and neurofilament. emNCSCs integrated into the *Ret.k^-^* intestinal wall and differentiated into neurons within the 7 days of culturing (Figure 11A). Because *Ret.k^-^* embryos totally lack the enteric nervous system in the small and large intestine [31], neurofilament-positive cells must be derived from injected human emNCSCs. Indeed, no staining for neurofilament was detected in the non-injected gut segments (Figure 11B). The endogenous enteric neurons were detected in the heterozygous animals (Figure 11C). The *Ret.k^-^* animals are unable to generate enteric neurons [31]. However, the *Ret.k^-^* intestines injected with human emNCSC show immunostaining for both GFP and neurofilament, likely due to the generation of neurofilament+ cells from human emNCSC (Figure 11D). These data suggest that emNCSCs are able to colonizing the *Ret.k^-^* gut and differentiating into neurofilament-positive cells (presumably enteric neurons).

### *In ovo* transplantation of emNCSCs

**Figure 12:**
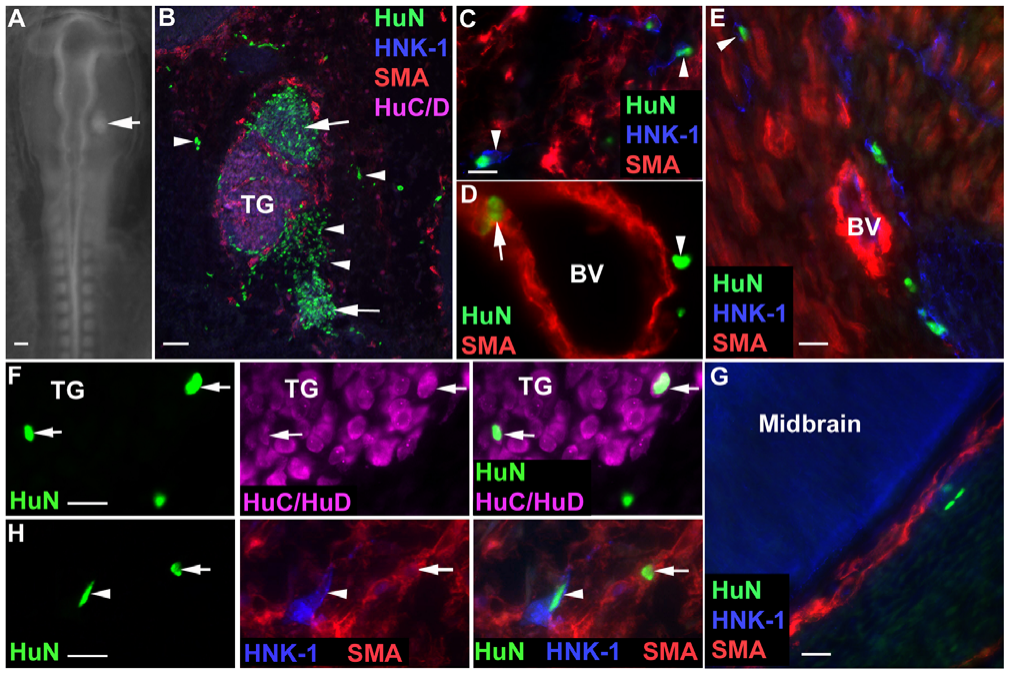
Injection of emNCSCs into the cranial NC pathway of chick embryos. (**A**) Whole mount view of a clump of grafted emNCSCs (arrow) injected into the cranial region of a stage 11 chick embryo. (**B–H**) Sections through the cranial region of chick embryos 5 days post-injection labeled with fluorescent antibodies to human nuclei (HuN, green), HNK-1 (blue), neuronal marker HuC/HuD (purple) and smooth muscle actin (SMA, red). (**B**) A number of human emNCSCs remain clustered and express HNK-1 (arrows) while individual cells that have left the graft site (arrowheads) are negative for HNK-1 and contribute to head mesenchyme. (**C**) emNCSCs along HNK-1-positive nerves (arrowheads). (**D**) SMA-positive (arrow) and -negative (arrowhead) emNCSCs around a blood vessel. (**E**) HNK-1-positive emNCSCs within skeletal muscle (arrowhead) do not express muscle actin (red). (**F**) Some emNCSCs (arrows, left and right panels) in the TG express the neuronal marker HuC/HuD (middle and right panels). (**G**) emNCSCs in undifferentiated mesenchyme near blood vessels around the perimeter of the midbrain. No emNCSCs were observed in the midbrain. (**H**) Differentiated emNCSCs (left and right panels) expressing SMA (arrow, middle and right panels) or HNK-1 (arrowhead, middle and right panels). BV - blood vessel; TG – trigeminal ganglion. Scale bars 200 µM in A, 100 µM in B, 20 µM in C–H.

Finally, we investigated the developmental potential of emNCSCs, namely, their ability to migrate, integrate and differentiate *in vivo*. GFP-labeled emNCSCs were injected into the chick embryo NC migration pathway beside the midbrain and rhombomeres 1 and 2 during the endogenous cranial NC migration (Figure 12A), and assayed for migration, appropriate targeting and differentiation. Cells were identified using immunohistochemistry of human nuclear antigen and markers for terminal differentiation. Five days after injection, transplanted cells were found in many of the destinations of cranial NC such as the branchial arches, the cranial ganglia and the head mesenchyme. Transplanted cells that migrated from the graft into the mesenchyme lost expression of HNK-1 (Figure 12B) and contributed to a variety of NC-derived tissues, both neural and mesenchymal. Some transplanted cells remained at the graft site and expressed low levels of HNK-1 but no neuronal markers (Figure 12B). Transplanted cells located throughout the cranial mesenchyme and along skeletal muscle fibers have elongated nuclei and expressed HNK-1, in this context, characteristic of Schwann cells (Figure 12C, E, H). Some transplanted cells were observed in undifferentiated cranial mesenchyme (Figure 12A, G), and among skeletal muscle fibers, potentially forming connective tissue. Many transplanted cells were located around blood vessels, some of which were positive for SMA (Figure 12D). emNCSCs were also seen differentiating into smooth muscle in other appropriate locations (Figure 12H). Numerous transplanted cells were found in the trigeminal and ciliary ganglia (Figure 12B and F), some of which expressed neuronal markers (Figure 12F) while others displayed glial morphology.

Both the location and the differentiation fates of transplanted cells were appropriate for cells of NC origin. In contrast, we failed to observe emNCSCs differentiating into cell types not normally derived from endogenous NC, such as neurons in the CNS. We concluded that emNCSCs transplanted in chick embryos during cranial NC migration respond to developmental cues, migrate along endogenous NC cells and differentiate into the appropriate tissues/structures.

## Discussion

**Figure 13:**
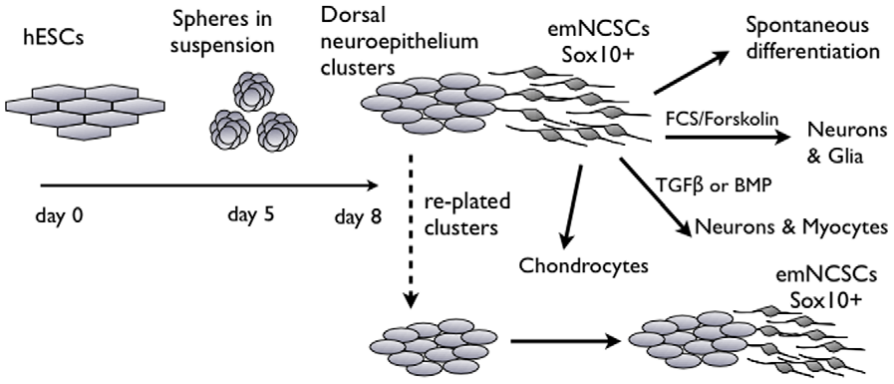
Scheme of early emNCSCs derivation from hESCs and differentiation into NC lineages. hESC clusters are suspended for 5 days in NS medium. On day 5 of suspension culture neurospheres were allowed to adhere on fibronectin-coated plates and cultured in NS medium for 3 more days. Adherent neurospheres composed of neuroepithelial rosettes are negative for Sox10 and CD73. Migrating cells are positive for the NC markers Sox10 and CD73 (13%). Both cell types are positive for p75 and HNK-1. Rosettes can be manually excised and replated (dotted arrow) to yield additional waves of Sox10-positive emNCSCs. The adherent rosettes are therefore competent to become NC after they have undergone the EMT. Migratory emNCSCs can be allowed to spontaneously acquire NC lineage fates or can be directed to enrich for certain cell fates by the addition of growth factors.

Here, we characterize a population of cells emigrating from hESC-derived adherent neurospheres plated on fibronectin and provide evidence that they mimic the dorsal neural tube containing premigratory NCSCs and resembling their site of origin *in vivo*. The adherent neurospheres are mainly composed of neuroepithelial rosettes, which express Pax3 and Sox9, markers of dorsal neuroepithelium and premigratory NC [55], [56], [57], but are negative for the ventral neural tube marker Nkx2.2 [58]. Cells that leave neuroepithelial clusters and start migrating on fibronectin uniformly upregulate Sox10, a critical transcription factor for NC development [22] (Figure 13). The clusters remain negative for Sox10 and can be mechanically isolated. After replating on fibronectin, they give rise to a second wave of emigrating cells, which also upregulate Sox10. Most of the cells within replated clusters remain negative for Sox10 3 days after replating. It is likely that virtually all cells within the original adherent neurospheres are competent to emigrate and upregulate Sox10. Indeed, over 80% of cells within adherent neurospheres are positive for the markers of dorsal neuroepithelium and lack the ventral markers, consistent with the cells being premigratory NC.

Analysis of global gene expression profile of cells within the clusters and emNCSCs revealed selective upregulation of the NC-related genes in the migratory cells. For example, homeobox gene *PhOX2b* is essential for the development of both autonomic and sympathetic neurons [59], [60] and regulates the dynamic expression of RGS4 [61]. The upregulation of *DLK1* and *HEY1* in emNCSCs is consistent with the role of Notch signaling in NC differentiation and maintenance [62]. We detected selective upregulation of gonadotropin-releasing hormone 2 (GnRH2) in emNCSCs. Indeed, some cells releasing GnRH arise from cranial NC [63] and their differentiation is modulated by the NC transcription factors *SOX10* and *FOXD3* [64]. It is tempting to speculate that these differences between the migratory cells and adherent clusters will provide insight into the genes and pathways involved in delamination of human NC.

In contrast to previous reports [15], a much larger fraction (∼100-fold increase) of emNCSCs is positive for the mesenchymal cell marker CD73, which is a unique characteristic of cephalic NC. Cephalic NC cells are known to emigrate earlier than trunk NC in all species studied, including recently derived human NC cells [40]. Previously described NCSCs lack the expression of connexin 43 ([15] supplementary microarray data), which could be due to the extensive passaging. In contrast, the emNCSCs described in this study uniformly express connexin 43, previously found to be characteristic of early migrating NC [44]. Finally, previous studies in chick demonstrated that BMP inhibition (*e.g.,* with Noggin) can block NC specification during early, but not late stages of NC development [23]. Treatment with Noggin quantitatively abolishes the appearance of emNCSCs. Taken together these data strongly argue that we have identified and characterized early migratory NCSCs, likely resembling cephalic NCSCs, derived from human ES cells.

EmNCSCs express the classical marker of NCSC, p75; however, we found that in our setting, dorsal neuroepithelium-like cells also express p75. Similarly, both cell types are positive for HNK-1, a well-known NC marker in avian but not murine embryos [47]. Indeed, at the time of NC emigration in mice the most dorsal part of the neural tube, including some premigratory NC cells residing in that area, are positive for p75 [39]. Similarly, human neural tube cells express p75 [40]. Previously, when hESCs were neuralized using stromal feeder cells [15], [16], only a proportion of cells in culture (*e.g.,* the most outer part of neuroepithelieal rosettes) were found positive for both p75 and HNK-1 [15]. The difference in the neuralization protocols is a likely explanation for the observed discrepancies. The heterogeneous p75 reactivity within neuroepithelial clusters was observed using three different antibodies; rabbit polyclonal serum and two mouse monoclonal antibodies (including one directly conjugated, previously used for isolation of human NCSCs [16].) For HNK-1 staining, we used the same monoclonal antibody staining conditions as previously reported [15].

Recent evidence in avian embryos suggests that the onset of NC specification occurs during gastrulation, where epiblast cells that express the transcription factor Pax7, are specified to form NC cells in the absence of exogenous factors but in a manner that requires Pax7 itself [19]. In the mouse NC, the Pax3 protein may be functionally equivalent to the Pax7 [46]. We observed early and robust upregulation of Pax3 during hESC neutralization, such that by day 5 of neuralization ∼80% of cells were positive for Pax3. Remarkably nearly 60% of the cells were positive for Sox9, a marker of premigratory NCSCs. These findings suggest that under current neuralization conditions most neural progenitors express markers associated with NC competence and specification. Despite their early specification at gastrula stages, NC cells do not delaminate from the neural tube until late neurulation and do not initiate differentiation until late migratory and post-migratory stages. Furthermore, execution of NC cell fates *in vivo* requires continuous Wnt signaling [65] while various growth factors appear to direct differentiation along distinct pathways [49], [66]. Wnts and BMPs mediate NC induction in several model species [6], [65], [67]. In a previous study using human cells, melanocytes were generated from hESCs using Wnt3a-conditioned media; however the presence of multipotent NCSCs was not demonstrated [68]. Indeed, microarray analysis of neurosphere cultures confirmed the upregulation of *Wnt1*, *Wnt3a*, *BMP4* and *BMP5*, as well as the expression of transcription factors downstream of Wnt and BMP signaling. EmNCSCs expressed several transcription factors characteristic of early NC specification in chick, mouse, and *Xenopus*. For instance, *MSX1* and *ZIC1*, two of the earliest NC genes, were upregulated 48 and 72 hours, respectively, after the start of neuralization. These two genes have been demonstrated to be pivotal in the development of the NC and the expression of later NC-specific genes [55], [69], [70], [71]. The later NC-specifying genes include *Sox9, Sox10* and *Snail*, which proceed to regulate the EMT and migration of NC cells from the neural tube [70], [72], [73]. Consistent with these data, expression of *Sox9*, *Sox10* and *SNAIL* was upregulated during neuralization of hESCs. The chronological order of upregulation of some NC genes in the hESC cultures may follow a different timetable compared to known *in vivo* models; whether this is due to an *in vitro* artifact or reflects species differences remains to be determined.

*In vivo,* the NC competency zone (area around the neuroectoderm border) is mediated by BMP and Wnt signaling with additional signals, including Wnts mediating the induction of the NC [52], [53], [74] and delamination of NC from the neural tube [75], [76]. Different from our approach, most protocols for human NC derivation from ES cells use exogenous BMP to facilitate the differentiation [12], [15], [77]. To test if BMP pathway is also important for the generation of emNCSCs, we treated neurosphere cultures with Noggin. After treatment with Noggin, the expression of NC markers Sox10, p75 and HNK-1 was nearly ablated compared to controls. Treatment with the Wnt/β-catenin antagonist Dkk1 resulted in the ablation of Sox10 and HNK1, but not p75. These results are consistent with the distinct mechanisms of action of Noggin and Dkk1. While Noggin (produced by the notochord *in vivo*) re-specifies the dorsal neuroepithelium into ventral fates, Dkk1 inhibits the early steps of NC induction, without altering the axial cell fates or general patterning [53]. In our hands, the dorsal neuroepithelium-like clusters (adherent neurspheres) are positive for p75. The loss of Sox10 and p75 after the addition of Noggin is consistent with re-specification of dorsal neuroepithelium into ventral neuroepithelium, which typically does not express NC markers. Following the same logic, the addition of Dkk1 may inhibit the NC specification, *i.e.* appearance of Sox10- and HNK-1-positive emNCSCs, but does not re-specify the dorsal neuroepithelium-like cells, which remain positive for p75. Indeed, the human neural tube cells were found positive for the p75 antigen at the time when the NC is generated [40]. It remains to be determined if this expression is localized to the premigratory neural crest in the dorsal part of human neural tube.

In addition to the patterning growth factors discussed above, matrix elasticity may affect hESC differentiation towards NC lineages, similar to that seen for muscle differentiation [78], [79]. It will be important to investigate hESC differentiation into emNCSC using various elasticity matrixes.

Finally, we assessed the ability of emNCSCs to migrate *in vivo,* incorporate into NC derivatives and differentiate appropriately. Grafted emNCSCs efficiently contributed to a variety of NC-derived tissues and differentiated appropriately. This finding demonstrates that emNCSCs are competent to contributing to NC derivatives such as the trigeminal ganglia, as cells that do not normally incorporate into the trigeminal ganglia are excluded from the developing ganglia even when they are immediately adjacent to it [80]. Furthermore, emNCSCs contributed to connective tissues including smooth muscle. Therefore, emNCSCs are capable of extensive migration to appropriate NC cell destinations and appear to have the ability to interact with adjacent host NC cells and differentiate efficiently compared to late emNCSCs, *in vivo* [15]. Critically, transplanted human emNCSCs do not contribute to the non-NC cell types, such as CNS tissue (*e.g.,* midbrain).

Given the fact that these cells are derived in an antigen free environment (no co-culture with murine tissue) the protocol for the derivation of emNCSCs is ideal for generating emNCSC-derived tissues in the culture dish that can subsequently be used for patient treatment (*e.g.,* neurocristopahties). To determine the therapeutic potential of these cells for treating a disease model, we investigated the ability of emNCSCs to colonize aganglionic embryonic guts in organotypic cultures. EmNCSCs were found to be capable of colonizing aganglionic guts and differentiating into neurofilament-positive cells, presumably enteric neurons, in *ex vivo* gut cultures. This suggests that emNCSCs might be useful in cell replacement therapies to treat neurocristopathies.

## Supporting Information

Figure S1 Molecular characterization of hESC neuralization. (A–D) Microarray profiles of hESC derived neruospheres over a 10-day (240 hour) time period. Markers are indicated in the figure. Y-axis shows the fold increase, X-axis shows the time (hours). (E) The Q-PCR profile for Pax3 induction, X-axis shows hours of induction (0-144), Y-axis indicates fold increase of Pax3.
(0.12 MB TIF)

Figure S2 Spontaneously differentiated emNCSC cultures. Spontaneously differentiated emNCSC cultures yield a mix of cell types: (A) SMA (green), Peripherin (red), (B) GFAP (green), and MAP2 (red) positive cells, (C) GFAP (red), and HNK1 (green) positive cells.
(0.70 MB TIF)
