# Supplementary figures and images for: Early Acquisition of Neural Crest Competence During hESCs Neuralization
Source: PLoS One. 2010 Nov 9;5(11):e13890. doi: 10.1371/journal.pone.0013890 (PMC2976694; doi:10.1371/journal.pone.0013890)

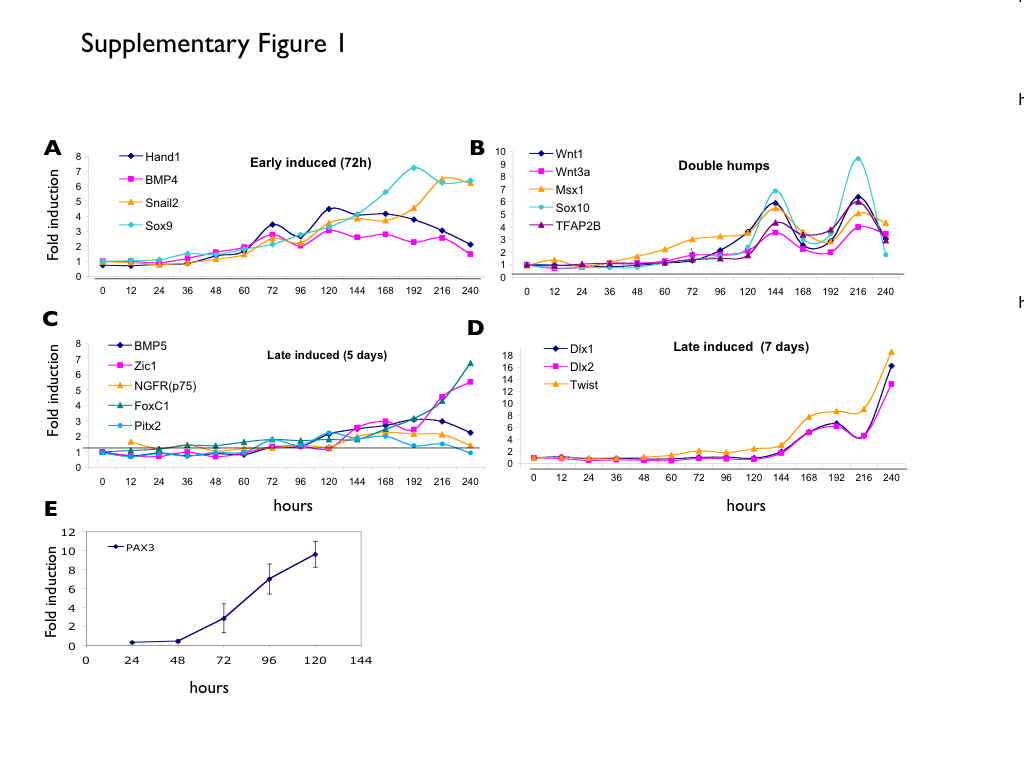

Supplement: Figure S1 — Molecular characterization of hESC neuralization. (A–D) Microarray profiles of hESC derived neruospheres over a 10-day (240 hour) time period. Markers are indicated in the figure. Y-axis shows the fold increase, X-axis shows the time (hours). (E) The Q-PCR profile for Pax3 induction, X-axis shows hours of induction (0-144), Y-axis indicates fold increase of Pax3. (0.12 MB TIF) [file pone.0013890.s001.tif]

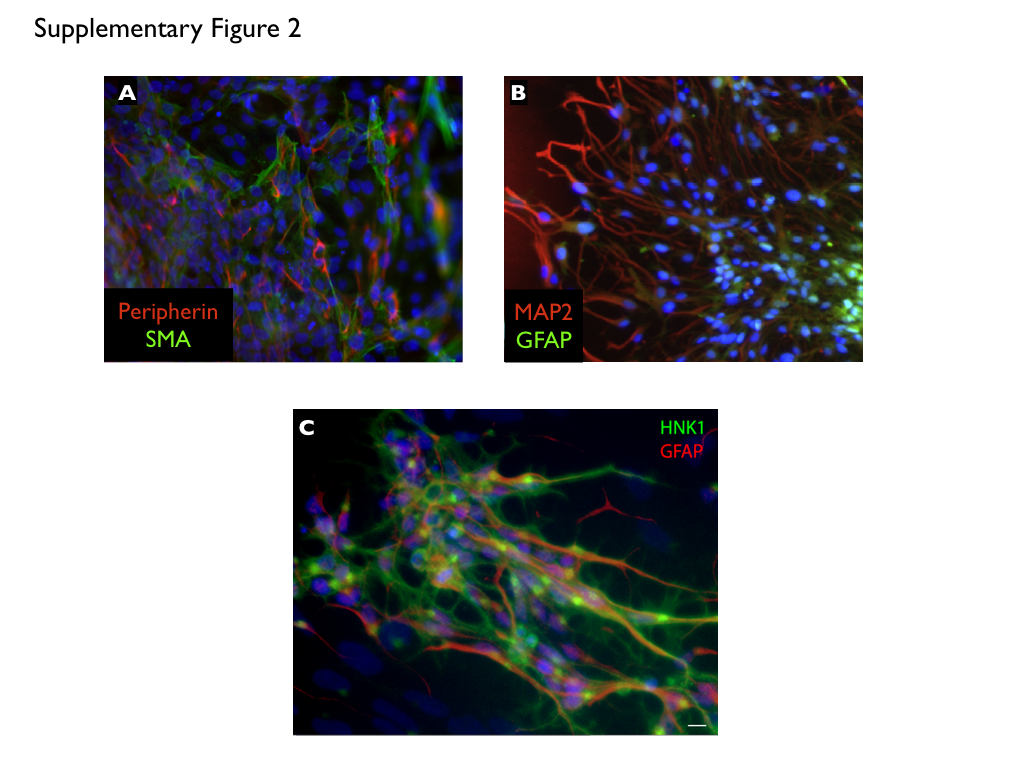

Supplement: Figure S2 — Spontaneously differentiated emNCSC cultures. Spontaneously differentiated emNCSC cultures yield a mix of cell types: (A) SMA (green), Peripherin (red), (B) GFAP (green), and MAP2 (red) positive cells, (C) GFAP (red), and HNK1 (green) positive cells. (0.70 MB TIF) [file pone.0013890.s002.tif]
